# Linking Cortical Morphometry in Self‐Limited Epilepsy With Centrotemporal Spikes to Cognition, Function, and Molecular Architecture

**DOI:** 10.1002/cns.70794

**Published:** 2026-02-23

**Authors:** Siqi Yang, Jie Xia, Wei Liao, Yimin Zhou, Chengzong Peng, Juan Wang, Zhiqiang Zhang

**Affiliations:** ^1^ School of Cybersecurity (Xin Gu Industrial College) Chengdu University of Information Technology Chengdu People's Republic of China; ^2^ School of Acupuncture and Tuina Chengdu University of Traditional Chinese Medicine Chengdu People's Republic of China; ^3^ The Clinical Hospital of Chengdu Brain Science Institute, School of Life Science and Technology University of Electronic Science and Technology of China Chengdu People's Republic of China; ^4^ Laboratory of Neuroimaging, Department of Radiology, Jinling Hospital Nanjing University School of Medicine Nanjing People's Republic of China

**Keywords:** brain heterogeneity, brain morphology, nonnegative matrix factorization, normative modeling, self‐limited epilepsy with centrotemporal spikes

## Abstract

**Aims:**

Self‐limiting epilepsy with centrotemporal spikes (SeLECTS) is the most common type of pediatric epilepsy, characterized by age‐dependent seizures, which usually occur during the development of a child's brain. This condition is associated with heterogeneous neurodevelopmental alterations, including cortical thinning, changes in subcortical structures, and atypical development linked to the disease.

**Methods:**

To establish an integrative model of neurodevelopment in SeLECTS, we investigated how its structural brain alterations are linked to clinical phenotypes, aberrant brain network function, and the local molecular architecture. Using normative modeling, we analyzed magnetic resonance imaging (MRI)‐derived morphometric features, specifically cortical thickness and subcortical volumes, in a multicenter preschool cohort (devCCNP, *n* = 457) and a SeLECTS cohort (*n* = 187) and generated deviation matrices specific to SeLECTS.

**Results:**

Nonnegative matrix factorization was applied to decompose these matrices into eight deviation components, revealing biologically interpretable patterns of heterogeneity, along with subject‐specific loadings that quantify the expression of these components in individual subjects. Behavioral partial least squares analysis identified significant associations between subject‐specific loadings and phenotypic profiles in SeLECTS, suggesting that factors such as age, medication history, and disease duration are important for morphological development—particularly in temporal and frontal regions associated with cognitive control and language. Furthermore, we explored the molecular basis of the morphometric deviation components by mapping their spatial expression to features related to functional cognition, neurotransmitter/transcript profiles, and mitochondrial characteristics.

**Conclusion:**

Collectively, this study provides a novel framework for elucidating the neuroanatomical heterogeneity of epilepsy, offering insights into its behavioral and molecular correlates.

## Graphical abstract

This study provides a novel framework modeling neurodevelopment in self‐limited epilepsy with centrotemporal spikes. We identify eight biologically interpretable brain structural deviation patterns, which are linked to clinical phenotypes and molecular profiles involving cognition and mitochondrial function. This integration elucidates multidimensional brain‐behavior‐molecular correlates in pediatric epilepsy.

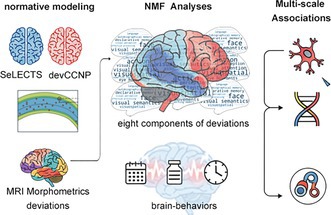

## 1 Introduction

Self‐limited epilepsy with centrotemporal spikes (SeLECTS), previously termed benign Rolandic epilepsy, is the most prevalent childhood epilepsy syndrome, accounting for 15%–25% of pediatric epilepsies [1]. The condition typically manifests between 3 and 14 years of age, with a peak incidence at 7–8 years, and most patients experience spontaneous remission before adolescence. Despite its “self‐limited” designation, individuals with SeLECTS often exhibit subtle but measurable cognitive, linguistic, and behavioral impairments during active epileptic periods [2, 3, 4]. These transient neuropsychological deficits are hypothesized to result from centrotemporal spike‐mediated disruptions in cortical developmental pathways.

Emerging evidence underscores significant interindividual neurodevelopmental heterogeneity in children with SeLECTS, which may modulate variations in clinical presentations, cognitive deficits, and long‐term outcomes. Electroencephalography (EEG), the primary diagnostic tool for SeLECTS, reveals considerable variability in centrotemporal spike distribution, frequency, and their associations with cognitive functions. Recent studies suggest that these EEG signal variations reflect underlying heterogeneity in functional neural networks [5]. Magnetic resonance imaging (MRI) studies have further elucidated neurodevelopmental divergence, demonstrating marked individual variations in structural architecture and functional connectivity, which may correlate with the severity of cognitive impairments [6, 7]. For instance, an EEG‐triggered fMRI study using single‐case analysis identified significant variability in spike wave‐related blood‐oxygen‐level‐dependent (BOLD) activation patterns in the centrotemporal region, suggesting that individualized neural network remodeling may account for the diversity of cognitive impairments [8]. These heterogeneous activation profiles are associated with domain‐specific cognitive deficits, including language processing and executive function, indicating that individualized neural circuit remodeling may underlie clinical manifestations. Although positron emission tomography (PET) is underutilized in SeLECTS research, preliminary findings suggest that individual metabolic signatures may localize epileptogenic foci and correlate with cognitive performance [9]. Collectively, these multimodal findings highlight three key dimensions of neurobiological heterogeneity—structural, functional, and metabolic—which may arise from polygenic predispositions, environmental factors, and subject‐specific neurodevelopmental trajectories.

Recent advances in integrative methodologies, combining normative modeling with nonnegative matrix factorization (NMF), offer promising tools for resolving individual‐level heterogeneity in neuropsychiatric disorders [10, 11]. This synergistic approach quantifies subject‐specific deviations by establishing population reference standards while decomposing heterogeneous disease patterns through data‐driven factorization [12]. Specifically, normative modeling constructs continuous distributions of brain features from healthy reference cohorts, enabling the quantification of individual deviations relative to typical developmental trajectories [13, 14]; NMF ensures nonnegativity and interpretability of decomposition results, making it well‐suited for analyzing complex imaging data [15, 16, 17]. The orthonormal projective variant of NMF (opNMF) further decomposes deviation matrices into dimensionally reduced components, each representing distinct patterns of abnormality, such as structural or functional alterations in specific brain regions. These components can be projected into brain space for visualization, elucidating the biological heterogeneity of the disease population, while subject‐specific loadings quantify individual contributions to these patterns. This integrated normative modeling‐NMF framework facilitates multimodal data fusion, advancing mechanistic insights into neurodevelopmental pathways and enhancing biological interpretability in children with SeLECTS.

This study investigates neurodevelopmental heterogeneity in SeLECTS using an integrated normative modeling and opNMF approach. Normative modeling was applied to MRI‐derived morphometric features, specifically cortical thickness and subcortical volumes, in a multicenter preschool cohort (CCNP, *n* = 457) and a SeLECTS cohort (*n* = 187). This process generated deviation matrices specific to SeLECTS, quantifying individual deviations from normative neurodevelopmental trajectories. Subsequently, opNMF was employed to decompose these deviation matrices into: (i) deviation components, which delineate biologically interpretable patterns of heterogeneity, and (ii) subject‐specific loadings, which quantify the expression of these components in individual participants. We hypothesized that morphometric deviation components in SeLECTS manifest as distinct, nonoverlapping, and spatially localized patterns. To test this, we examined the correspondence between subject‐specific loadings and individual phenotypic profiles. Additionally, we explored the molecular underpinnings of these morphometric deviation components by mapping their spatial expression to features associated with functional cognition, neurotransmitter and transcript profiles, and mitochondrial characteristics.

## 2 Methods

### 2.1 Subjects

The SeLECTS cohort comprised 187 children with epilepsy (98 boys and 89 girls, age range: 4–18 years; mean ± SD: 9.09 ± 2.66 years). Additionally, the study included 108 age‐ and sex‐matched healthy controls (61 boys, 47 girls; age range: 4–15 years; mean ± SD: 9.42 ± 2.49 years) (Table 1). This study exclusively enrolled subjects who exhibited unremarkable findings on conventional (structural) MRI, a criterion defined by the absence of evidence for brain tumors, vascular malformations, cavernous malformations, focal cortical dysplasia, hippocampal sclerosis, previous stroke, or any other space‐occupying lesions considered to be potentially epileptogenic. Epilepsy diagnoses were confirmed by two board‐certified neurologists according to the International League Against Epilepsy (ILAE) classification [1]. Routine clinical EEG findings confirmed the typical pattern for SeLECTS, characterized by slow, biphasic, and high‐voltage interictal epileptiform discharges (spike–wave complexes) that were primarily localized to the centrotemporal (Rolandic) regions, with a subset of participants exhibiting spikes that spread to adjacent frontal or parietal areas. The study protocol complied with the Declaration of Helsinki (1975) and was approved by the Medical Ethics Committee of Jinling Hospital, Nanjing University Medical School. Written informed consent was obtained from all subjects or their parents. Exclusion criteria included the presence of metal implants, a history of severe neurological or psychiatric disorders, or other clinically significant illnesses. Additional exclusions comprised inability to comply with or comprehend the study protocol, as well as any contraindications for MRI procedures. All normal participants had no history of neurological disorder or psychiatric illness and no gross abnormalities on brain MRI images. Neuropsychological assessments were administered to a subset of 75 children with SeLECTS, including the Raven's Standard Progressive Matrices (RSPM)—a nonverbal tool designed to assess analog perception, reasoning, and abstract abilities, and IQ scores were used to quantify the test results in the current work. Other assessments include the Integrated Visual and Auditory Continuous Performance Test (IVA‐CPT), which is primarily used to screen for attention deficit hyperactivity disorder, has a total of 10 subdimensions, including: hyperactivity, cautious, consistency, perseverance, alertness, focus, speed, balance, agility, sustainability and sensorimotor. These dimensions are ultimately summarized into three aspects: full‐scale comprehension, full‐scale control, and full‐scale attention (see Table S1).

**TABLE 1:** Demographic and clinical characteristics of subjects.

Demographics	SeLECTS cohort		SeLECTS vs. HC		CCNP		SeLECTS cohort vs. CCNP	
	SeLECTS (*n* = 203)	HC (*n* = 254)	Statistics	*p*	CKG (*n* = 203)	PEK (*n* = 254)	Statistics	*p*
Sex (males/females)	98/89	61/47	0.46^b^	0.50	97/106	144/110	0.10^b^	0.76
Handedness (left/right)	0/187	1/107	1.74^b^	0.19	2/201	7/247	3.63^b^	0.06
Age (years)	9.09 ± 2.66	9.42 ± 2.49	9284^a^	0.25	11.74 ± 3.14	9.41 ± 2.68	38,771^a^	**< 0.01**
Duration of illness (months)	19.85 ± 21.22^c^ (*n* = 167)	—	—	—	—	—	—	—
Duration of medicine	17.87 ± 17.68^c^ (*n* = 103)	—	—	—	—	—	—	—
Dose of medicine	0.78 ± 0.78^c^ (*n* = 103)	—	—	—	—	—	—	—
Seizure frequency (per month)	5.29 ± 8.66^c^ (*n* = 167)	—	—	—	—	—	—	—

*Note*: The bold values indicate that the *P* value is lower than 0.05, which is statistically significant.

^a^ Represented Mann–Whitney *U* test.

^b^ Represented Chi‐square test.

^c^ Indicates that there is missing data in the corresponding variable.

To evaluate cortical morphometric deviations in children with SeLECTS, a normative modeling framework was established using an age‐specific developmental dataset derived from the developing Chinese Color Nest Project (devCCNP; https://ccnp.scidb.cn). The dataset comprised two independent cohorts: CKG (*N* = 203) and PEK (*N* = 254), consisting of typically developing Chinese school‐aged children and adolescents (241boys, 216 girls; age range: 3–18 years; mean ± SD: 10.44 ± 3.11 years) (Table 1). All subjects were free of neurological or psychiatric disorders, with no structural abnormalities detected on brain MRI. The study protocol received ethical approval from the Institutional Review Board of the Institute of Psychology, Chinese Academy of Sciences. Written informed consent was obtained from legal guardians, with additional written assent provided by subjects aged ≥ 8 years.

### 2.2 MRI Acquisition

Structural MRI data were acquired on a Siemens Trio Tim 3.0‐Tesla scanner at Jinling Hospital. Head movement was minimized using foam padding, with all subjects instructed to maintain closed eyes and head immobility during scans. High‐resolution T1‐weighted images were obtained in the sagittal plane using a magnetization‐prepared rapid gradient‐echo (MPRAGE) sequence with the following parameters: repetition time/echo time (TR/TE) = 2300/2.98 ms, flip angle = 9°, field of view = 256 × 256 mm^2^, matrix size = 256 × 256, slice thickness = 1 mm, no interslice gap, and 176 slices.

Additional T1‐weighted MRI data were derived from the devCCNP, whose full technical specifications have been previously published [18]. Structural MRI data of the CKG sample were collected using a 3.0‐T Siemens Trio MRI scanner at the Center for Brain Imaging, Southwest University. Participants were asked to keep their eyes closed to rest. High‐resolution T1‐weighted images were obtained in the sagittal plane using a 3D MPRAGE sequence with the following parameters: TR/TE = 2600/3.02 ms, flip angle = 8°, field of view = 256 × 256 mm^2^, matrix size = 256 × 256, slice thickness = 1 mm, and 176 slices. The PEK sample was imaged on a 3.0‐T GE Discovery MR750 scanner at the Magnetic Resonance Imaging Research Center of the Institute of Psychology, Chinese Academy of Sciences. T1‐weighted images were obtained in the sagittal plane using a 3D SPGR sequence with the following parameters: TR/TE = 6.7/2.9 ms, flip angle = 12°, field of view = 256 × 256 mm^2^, matrix size = 256 × 256, slice thickness = 1 mm, and 176 slices.

### 2.3 Data Preprocessing

Regional‐wise cortical thickness (CT) and subcortical volume (SV) for each subject were estimated automatically as part of the following processing, in both the SeLECTS cohort and the devCCNP datasets. Specifically, structural MRI processing pipelines were primarily utilized FreeSurfer [19], involving cortical segmentation and surface‐based reconstruction. Native‐space anatomical surfaces were generated from individual T1‐weighted images using FreeSurfer's *recon‐all* command. Morphometric estimates included CT across 68 cortical regions and SV in 14 subcortical structures. CT and SV were specifically selected as morphometric indices due to their demonstrated reliability and measurement stability across longitudinal assessments [20, 21].

### 2.4 Identifying Deviation Components and Subject‐Specific Loadings With NMF

**FIGURE 1:**
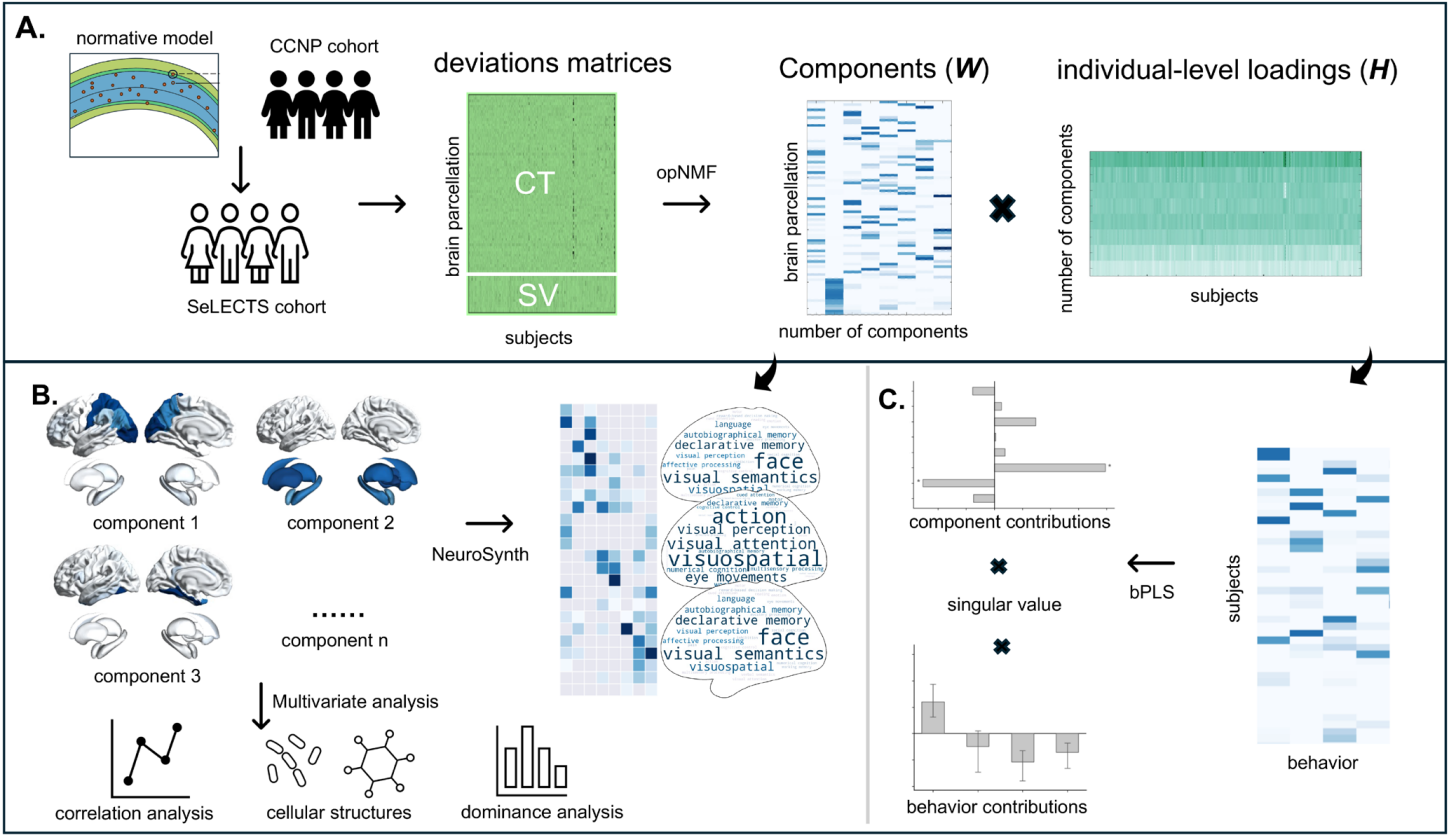
Workflow of the study. (A) A normative model was trained using cortical thickness (CT) and subcortical volume (SV) data from the CCNP cohort to estimate morphometric deviations in the SeLECTS cohort. Optimal nonnegative matrix factorization (opNMF) decomposed the deviation matrix into a component matrix (*W*), representing spatial attributes, and a loading matrix (*H*), capturing subject‐specific loadings for each component. (B) Spatial components were mapped to cognitive functions, neurotransmitter profiles, and mitochondrial characteristics. NeuroSynth meta‐analysis was performed to identify associations between component spatial patterns and psychological terms. Multivariate regression analysis assessed relationships between components and molecular properties (neurotransmitter receptors/transporters and mitochondrial features), with dominance analysis quantifying the contribution of each variable to the regression model. (C) Behavioral partial least squares (bPLS) analysis was conducted on subject‐specific loadings to identify covariant patterns between brain morphometry and clinical features, along with their respective contributions.

An overview of the analysis workflow can be found in Figure 1. Briefly, we first used the CCNP cohort to train the normative model to predict the morphometric deviations of the children with SeLECTS. The process of deviation prediction using the normative model is consistent with our previous work [13], referring to the framework of CentileBrain [14]. Specifically, after obtaining the CT and SV matrix from the devCCNP, we developed a normative model using sex and age as covariates. Leveraging the CentileBrain pipeline, we opted for multivariable fractional polynomial regression, incorporating the optimal covariate combination, which included site harmonization [22], as well as mean cortical thickness and total intracranial volume in the models for CT and SV regional measures, respectively. This benchmarking study demonstrated that a key advantage of using ComBat‐GAM is that it removes the need for model recalibration or parameter adaptation when applying the model to data from a new site, allowing the harmonized model to be transferred robustly and directly. Subsequently, sex‐ and region‐specific parameters from the normative model were applied to each regional morphometric measurement in the SeLECTS cohort. Normative deviations for each measurement were quantified as the ratio of the residual between observed measurements and model‐predicted values to the root mean squared error (RMSE) of the normative distribution. The conventional *Z*‐score is replaced because normative deviations use a personalized model that accounts for covariates like age and sex, transforming “normal” from a fixed global standard into a dynamic, context‐aware benchmark, leading to more sensitive and specific detection of disease‐related deviations in brain imaging. To verify the robustness of the trained model's performance, we evaluated the prediction error of the trained normative model on the healthy control group of the SeLECTS dataset (Methods in Figures S1 and S2).

To identify spatial patterns of neuroanatomical covariance, we applied the orthonormal projective extension of nonnegative matrix factorization (opNMF) [23, 24], to our cortical morphometric metrics (Figure 1A). The opNMF was an unsupervised multivariate statistical technique that decomposes the morphometric matrix (denoted as *X*_*m*×*n*_) into a component matrix (*W*_*m*×*k*_) and a loading coefficient matrix (*H*_*k*×*n*_), minimizing the reconstruction error between original *X* and reconstructed *WH* inputs. Importantly, *W* was initialized via nonnegative double singular value decomposition, enhancing the sparsity of the output component matrix. The NMF decomposition yields *k* components, each capturing latent covariance patterns underlying the input data. Specifically, *W* contains information regarding the spatial properties of each component, while the entries of *H* are loadings that specify the strength of expression of the component in each individual metric. In the current study, the rows of the input *X* corresponded to the 82 regional measures (68 for CT and 14 for SV), considered for analysis across the whole brain, while the columns comprised the CT or SV normative deviations for all 187 patients with SeLECTS. The implementation of the opNMF reference [25] (https://github.com/asotiras/brainparts).

To determine the optimal components (*k*), we assessed stability coefficient and reconstruction error gradients, the former quantified incremental accuracy gains with increasing *k* and the latter measured decomposition stability at a given *k*. To this end, we randomly split the cohort into two subsets, each subsample underwent independent opNMF, generating distinct component matrices *W*_A_ and *W*_B_. Firstly, the spatial similarity between component sets is quantified by calculating the cosine similarity matrix between the row vectors of *W*_A_ and *W*_B_, and then the decomposition stability is indexed by the average Pearson correlation coefficient of the paired components. The above procedure was repeated for 100 randomly half‐split for *k* = 2 to *k* = 20. To estimate the accuracy of a given *k*, we calculated the variation of the reconstruction error between *k*‐2 and *k*, and took the average of the differences in the data to report the gradient of the reconstruction error at the given *k*.

### 2.5 Cognitive Functional Decoding of the Morphometric Deviation Components

To relate the components of morphometric deviations with functional cognition, we focused on how the gradients could be related to cognitive terms using the NeuroSynth meta‐analytic database [26] (Figure 1B). Specifically, each component as an input image is associated with the association test meta‐analysis maps of the physical terms in the NeuroSynth database, and the output of the analysis is the z‐score related to the feature. Feature terms were derived from the 50‐set topic implemented by a previous study [27]. Of the 50, 30 were above the threshold of *z*‐statistic > 3.1, and 6 were removed as “noise” terms because they did not capture any coherent cognitive function, leaving 24 topic terms.

### 2.6 The Relationships Between Morphometric Deviation Patterns and Behaviors

To relate subject‐specific component loadings to the individual behavioral data, we performed behavioral partial least square analysis (bPLS) (Figure 1B). bPLS is an unsupervised multivariate technique that identifies patterns of variables that maximally covary across two sets of data through latent variables (LV). In our case, we utilized this technique to assess the patterns of associations across subject‐level NMF loadings (*H′* matrix) of cortical morphometric deviations and behavioral data (*Y*_*n*×*p*_ matrix, here, the dimensions of *p* was four, including age, sex, duration of disease, and seizure frequency) through LV. bPLS performs singular value decomposition on the obtained correlation matrix *H'Y* such that *H'Y* = *USV′*. Each output LV contains a pair of left/right singular vectors (from matrices *U* and *V*) and a singular value (from *S*). The left singular vector weights the original brain structure variables, while the right weights the original behavior variables, jointly constituting the extracted multivariate patterns. The singular value is proportional to the covariance between the brain morphometric features captured by the potential variable and the behavioral variable [28]. Therefore, each output LV describes the association between the individual‐level NMF loadings of morphometric deviations and behavioral data.

To evaluate the statistical significance of each LV, we adopted the permutation test. Specifically, the rows of the subject‐specific loading matrix were randomly shuffled 10,000 times, and bPLS was performed using the permutation loading brain matrix and the behavior matrix, which produced a distribution of singular values under the null hypothesis. We use the zero distribution to estimate the nonparametric *p* of each LV observed in the original data, with significance set to *p* < 0.05.

To evaluate the contribution of each behavioral metric or component to an LV, we adopted bootstrap resampling. The subject‐specific loading matrix (H′) and behavioral matrix (*Y*) were randomly sampled with replacement 10,000 times, and bPLS was performed on the data generated from each resampling to evaluate the sampling distribution of the weight coefficients of each variable under each LV. The contribution of components or behavioral variables to an LV was explained by dividing the true singular value by the standard error estimated by the bootstrap resampling as the bootstrap ratio (BSR). The higher BSR indicated the greater the contribution of this component variable. BSR values were thresholded at ±1.96, corresponding to a *p* = 0.05.

It should be noted that for this part of the study, the final sample for the primary bPLS analysis examining brain‐behavioral relationships comprised 38 participants. This reduction from the initial cohort was due to missing neuropsychological data—primarily resulting from participant noncompliance with the comprehensive assessment protocol—and missing medication details due to inaccurate recall. Our analysis indicated no significant differences in clinical characteristics (age, sex, disease duration, seizure frequency and medication status) between the included SeLECTS children and those excluded because of missing data. Furthermore, the term behavior was conceptualized as a multidimensional construct encompassing two distinct domains: (1) objective clinical disease burden, and (2) subjective cognitive performance, as evaluated by standardized neuropsychological tests. To elucidate their potentially distinct neurobiological correlates, we supplemented the primary analysis with focused bPLS investigations into brain‐cognition relationships (including a total of 69 subjects) and brain‐clinical variable relationships (including a total of 103 subjects).

### 2.7 Molecular Fingerprints Underpinning Morphometric Deviation Patterns

#### 2.7.1 Neurotransmitter Receptor Dataset

Given that neurotransmitter plays an important role in SeLECTS, we next sought to quantify the associations between receptors/transporters and the morphometric deviation components. Based on publicly accessible Positron Emission Tomography (PET) tracer images from over 1200 healthy participants across multiple studies [29], whole‐brain volumetric neurotransmitter receptor densities were assessed (https://github.com/netneurolab/hansen_receptors). The analyses encompassed 19 receptors/transporters constituting nine neurotransmitter systems: dopamine (D1, D2, DAT), serotonin (5‐HT1A, 5‐HT1B, 5‐HT2, 5‐HT4, 5‐HT6, 5‐HTT), norepinephrine (NET), glutamate (NMDA, mGluR5), GABA (GABAA), acetylcholine (α4β2, M1, VAChT), histamine (H3), opioid (MOR), and endocannabinoid (CB1). Cortical thickness and subcortical volume data were spatially coregistered onto PET image spaces.

#### 2.7.2 Mitochondrial Dataset

Given that mitochondrial oxidative phosphorylation drives brain activity and its dysfunction is implicated in neuropsychiatric disorders [30], we further determined the relationship between deviation components and mitochondrial features. Mitochondrial functional phenotyping was assessed through three key indicators reflecting energy transduction capacity: Complex I (CI), Complex II (CII), and Complex IV (CIV) activities representing oxidative phosphorylation enzymatic functions; Mitochondrial density (MitoD) quantified via citrate synthase activity and mtDNA content; Total respiratory capacity (TRC) and maximal respiratory capacity (MRC) characterizing integrated mitochondrial respiration. The MitoBrainMap mitochondrial phenotyping atlas is accessible at http://humanmitobrainmap.bcblab.com. This dataset provides six mitochondrial features (CI, CII, CIV, MitoD, TRC and MRC) that may facilitate correlative analyses with neuroimaging data. The analytical procedures examining associations between deviation components and mitochondrial features were aligned with those adopted for neurotransmitter receptors. Multivariate linear modeling was implemented to assess correlations, while dominance analysis was employed to quantify the relative contribution to each component.

### 2.8 Dominance Analysis

A multivariate linear regression modeling was subsequently employed to investigate associations between neurotransmitter receptor densities/mitochondrial features with morphometric deviation components, with dominance analysis performed to determine the relative contributions of each component [31] (https://github.com/dominance‐analysis/dominance‐analysis). The statistical significance of each model fit was evaluated through FDR‐corrected against spatial autocorrelation‐preserving null models (brainsmash). Each model was subsequently validated using distance‐dependent cross‐validation techniques. This method selects the nearest 25% of brain regions relative to the source region as the training set, with the remaining 75% serving as the test set. This process was repeated (for 100 iterations), with each brain region serving as the source region. The evaluation was performed by correlating the predicted deviation components with the empirical deviation components within the test set.

### 2.9 Null Models

We corrected the spatial autocorrelation using BrainSMASH (https://brainsmash.readthedocs.io/en/latest/), a brain proxy map with autocorrelation spatial heterogeneity [32]. In BrainSMASH, spatial autocorrelation in brain maps is operationalized through the construction of a variogram. The variogram quantifies, as a function of distance *d*, the variance between all pairs of points spatially separated by *d*. To generate spatial autocorrelation‐preserving surrogate brain maps, BrainSMASH produces random maps whose variograms are approximately matched to a target brain map's variogram.

## 3 Results

### 3.1 Spatial Patterns of Morphometric Deviations and Subject‐Specific Loadings

The results of the optimal nonnegative matrix factorization (opNMF) component analysis are presented in Figure S3. The stability coefficient exhibits a slight decline beyond *k* = 8, while the gradient of the reconstruction error stabilizes at higher resolutions after *k* = 8. These findings suggest that *k* = 8 captures the most prominent patterns in the input data, with diminishing returns in reconstruction accuracy for increased complexity. Consequently, *k* = 8 was selected for subsequent analyses.

**FIGURE 2:**
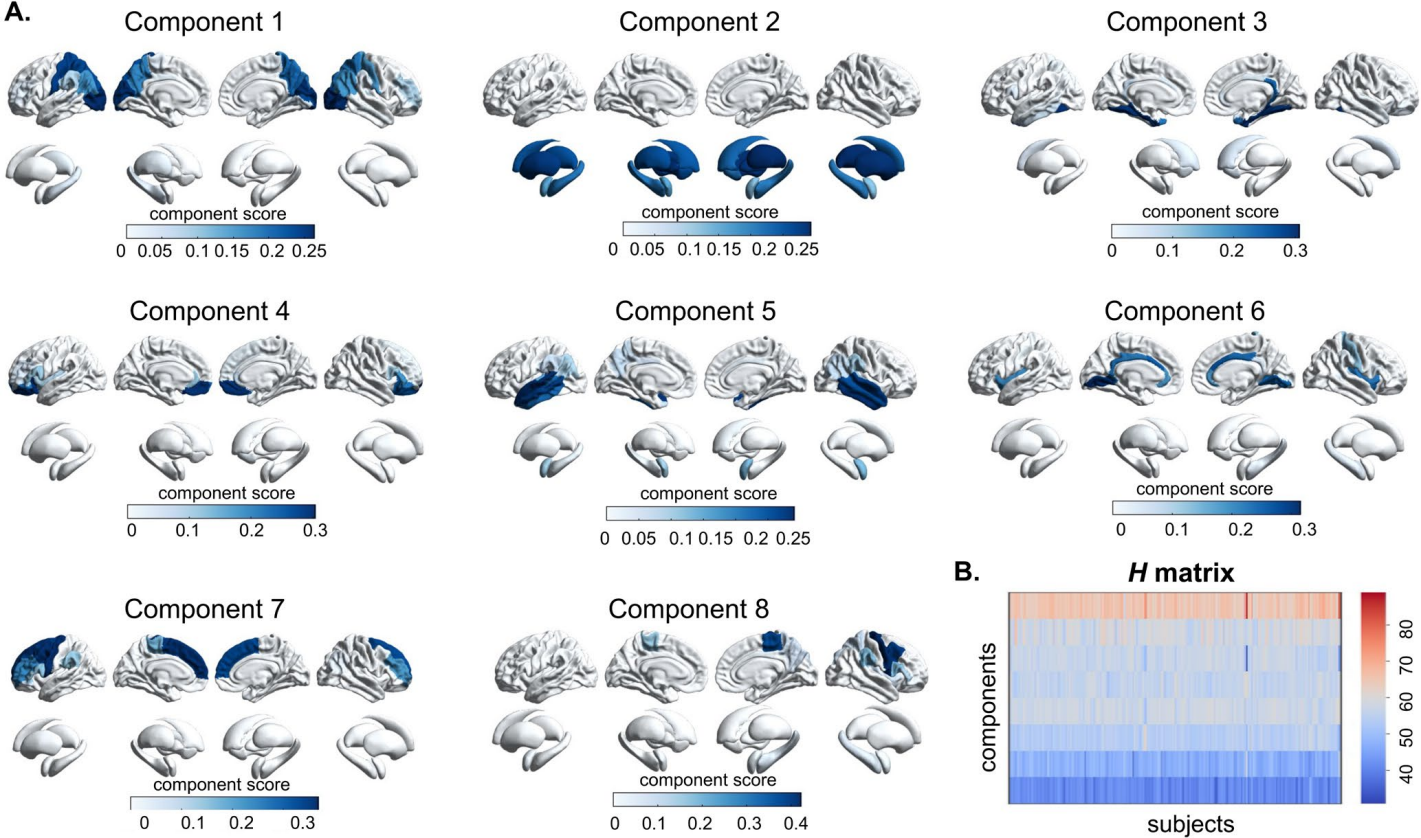
Distributions of eight components and individual‐specific loadings of morphometric deviation patterns. (A) Spatial maps of the eight components derived from opNMF. Darker blue indicates a greater contribution of a brain parcellation to the component. (B) Subject‐specific loadings for each component (*H* matrix), with rows representing components and columns representing subjects.

The eight opNMF components delineate distinct spatial patterns of morphometric normative deviations in the SeLECTS cohort (Figure 2A). Component 1 corresponds to the posterior brain regions, spanning the postcentral gyrus to the occipital lobe, and includes the left hippocampus and thalamus. Component 2 is primarily associated with all subcortical structures. Component 3 corresponds to the inferior temporal gyrus, caudate nucleus, and nucleus accumbens. Component 4 is linked to the inferior frontal gyrus. Component 5 encompasses the temporal lobe, supramarginal gyrus, left hippocampus, right caudate, and bilateral amygdala. Component 6 includes the insula, cingulate gyrus, and right hippocampus. Component 7 spans anterior regions, from the precentral gyrus to the inferior frontal gyrus. Component 8 is associated with the central sulcus, bilateral amygdala, and right hippocampus.

The *H* matrix effectively captures subject‐specific morphometric deviations relative to each spatial pattern, as compared to the normative deviation matrix. As shown in Figure 2B, each row represents a component, and each column corresponds to a subject. The entries in the *H* matrix quantify the contribution of each subject to a specific component. A high weight for Component 1, for instance, indicates a substantial contribution from a subject to the regions associated with that component through additive reconstruction of morphometric normative deviations. Overall, the contribution gradually decreases from Component 1 to Component 8.

### 3.2 Associations With Morphometric Deviation Patterns and Behavioral Variables

**FIGURE 3:**
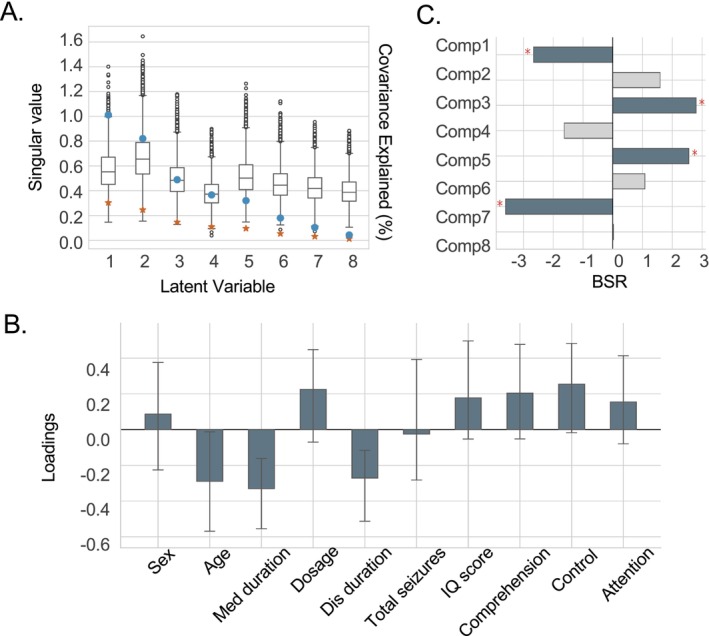
Latent morphometric‐behavioral relationships Identified by bPLS Analysis. (A) Scree plot of the singular value (blue dot) and covariance explained (red asterisk) by all latent variables (LVs) in the bPLS analysis, with LV1 significant at *p* = 0.01. (B) Bar chart illustrating the loadings of behavioral variables contributing to LV1. Bar height represents the loading magnitude, with error bars indicating the 95% confidence interval derived from bootstrap resampling. Blue bars denote statistically significant features. (C) Bar plot of bootstrap ratios (BSRs) for each component. Components with BSRs exceeding 1.96 (*p* < 0.05) are marked with a red asterisk.

We employed behavioral partial least squares (bPLS) analysis to investigate the associations between subject‐specific loadings and individual behavioral data. The bPLS analysis identified one significant latent variable (LV1; *p* = 0.01), which accounted for 30.3% of the covariance between subject‐specific loadings and behavioral variables. A scree plot illustrating the covariance of each identified latent variable is presented in Figure 3A. The singular values, variance interpretation, and corrected statistical values of the remaining variables can be found in the Table S2. The behavioral data contributing to LV1 included sex (*r* = 0.09, 95% confidence interval (CI) [−0.23, 0.37]), age (r = −0.29, CI [−0.57, −0.01]), duration of medication (*r* = −0.33, CI [−0.55, −0.16]), dosage (r = 0.23, CI [−0.07, 0.45]), duration of disease (r = −0.27, CI [−0.51, −0.12]), and total seizures (*r* = −0.03, CI [−0.28, 0.39]), IQ score (*r* = 0.18, CI [−0.05, 0.50]), full‐scale comprehension (*r* = 0.20, CI [−0.05, 0.48]), full‐scale control (*r* = 0.25, CI [−0.02, 0.48]), and full‐scale attention (r = 0.16, CI [−0.08, 0.41]) as shown in Figure 3B. Components significantly contributing to LV1 were Component 1 (posterior cortical regions, left hippocampus, and thalamus; bootstrap ratio [BSR] = −2.66, *p* = 0.01), Component 3 (the inferior temporal gyrus and caudate nucleus; BSR = 2.80, *p* = 0.01), Component 5 (temporal lobe, supramarginal gyrus, and bilateral amygdala; BSR = 2.56, *p* = 0.01), and Component 7 (anterior regions, spanning the precentral to inferior frontal gyrus; BSR = −3.60, *p* = 0.001), as depicted in Figure 3C.

Furthermore, we analyzed the brain‐cognitive relationship, which includes the RSPM and subcomponents of IVA‐CPT. The bPLS analysis identified one significant latent variable (LV2; *p* = 0.02), which accounted for 25% of the covariance between subject‐specific loadings and neuropsychological test scores (Figure S4). Detailed statistical outputs—such as singular values, explained variance, and variable‐specific *p*‐values—are provided in Table S3. We also performed a separate bPLS analysis to assess the associations between brain and clinical variables. This analysis also yielded one significant latent variable (LV1; *p* = 0.02), which explained 33% of the covariance between subject‐specific loadings and clinical measures (Figure S5 and Table S4).

### 3.3 Meta‐Analytic Functional Decoding of Components

**FIGURE 4:**
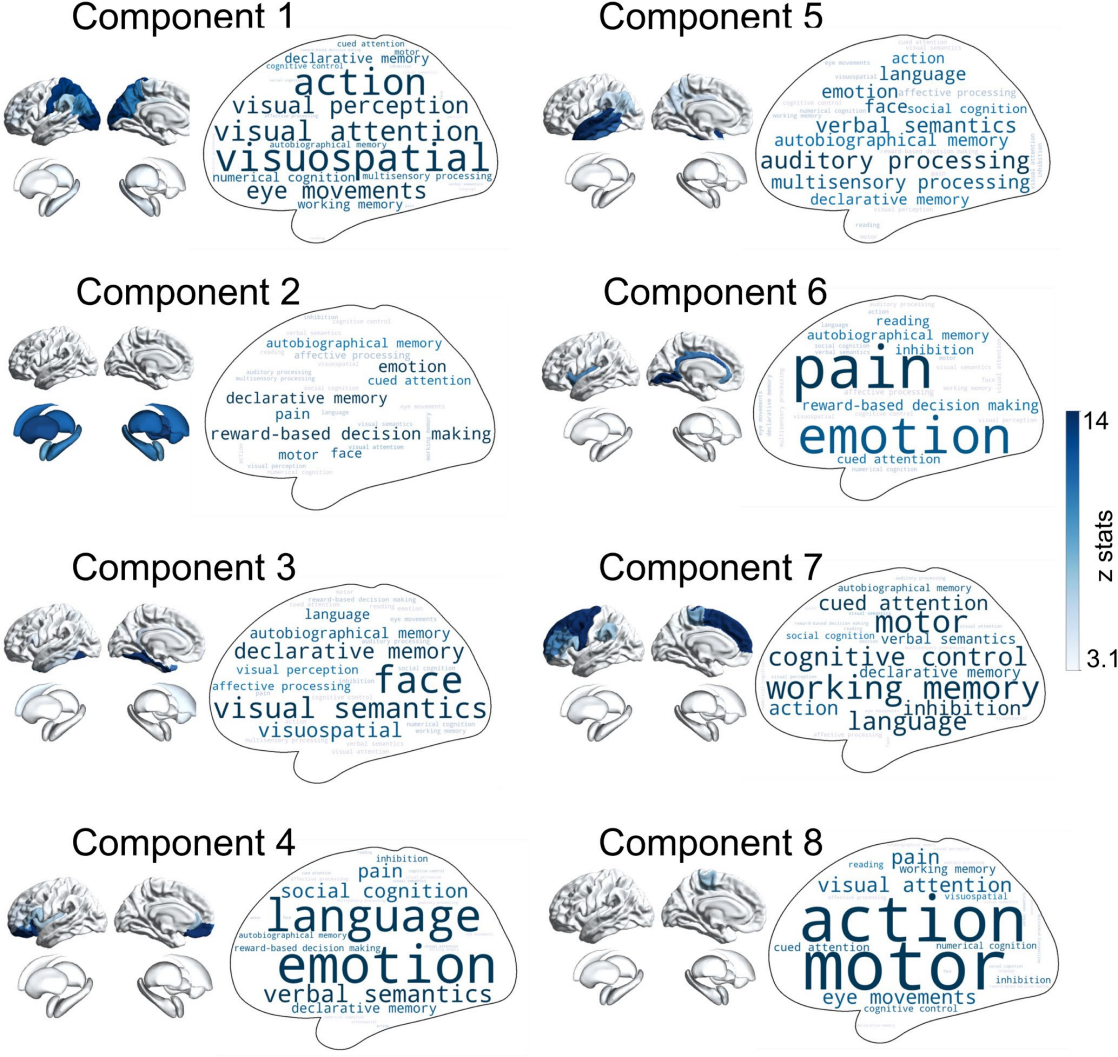
NeuroSynth meta‐analytic decoding of components. Word clouds represent psychological terms associated with each component, with corresponding functional maps correlated to component spatial patterns. Font size reflects the strength of the Pearson correlation between NeuroSynth term maps and component maps.

To elucidate the functional associations of each morphometric deviation component, we performed a meta‐analysis using NeuroSynth [26]. The psychological terminology most relevant to each component is depicted in Figure 4. The analysis revealed distinct functional domains associated with morphometric normative deviations in the SeLECTS cohort, summarized as follows: Component 1, visuospatial function; Component 2, emotion and decision‐making; Component 3, face and visual semantics; Component 4, emotion and language; Component 5, auditory processing and verbal semantics; Component 6, pain and emotion; Component 7, working memory and cognitive control; and Component 8, action and motor function.

### 3.4 Molecular Fingerprints Underpinning Morphometric Deviation Patterns

**FIGURE 5:**
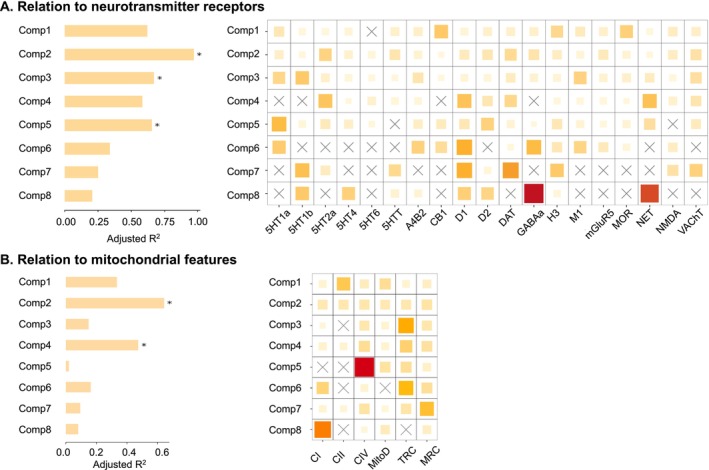
Associations between the morphometric deviation component and the molecular profiles. (A) Significance and relative contributions of neurotransmitter receptors/transporters to morphometric deviation components. (B) Significance and relative contributions of mitochondrial features to morphometric deviation components. Model significance was evaluated using a spatial autocorrelation‐preserving null model, corrected for multiple comparisons (false discovery rate, FDR). Asterisks indicate significant models (*p*_FDR_ < 0.05). Dominance analysis partitioned the adjusted *R* ^2^ among predictor variables, quantifying each variable's relative contribution. The contribution percentage is calculated as the variable's dominance normalized by the total model fit (adjusted *R* ^2^). Darker cell colors indicate higher contributions of individual predictors (receptors/transporters or mitochondrial features).

To examine the relationship between SeLECTS deviation components and neurotransmitter receptors/transporters, we fitted a multiple regression model to predict the spatial patterns of morphometric deviation components from the distribution of neurotransmitter receptors and transporters (Figure 5A). The model was cross‐validated using a distance‐dependent approach Figure S6. A strong correspondence was observed between the deviation components and receptor density, with adjusted *R* ^2^ values of 0.97 for Component 2 (*p*_FDR_ = 0.0008), 0.67 for Component 3 (*p*_FDR_ = 0.02), and 0.58 for Component 5 (*p*_FDR_ = 0.04). These findings suggest that the overlapping spatial topography of multiple neurotransmitter systems may contribute to SeLECTS‐specific deviation patterns. Dominance analysis identified the serotonin receptors 5‐HT2a, 5‐HT1b, and 5‐HT1a as the primary contributors to the spatial patterns of Components 2, 3, and 5, respectively.

To investigate the association between SeLECTS deviation components and mitochondrial characteristics, we fitted a multiple regression model to predict their spatial relationships (Figure 5B). The model was cross‐validated using a distance‐dependent approach (Figure S7). A significant fit was observed between the deviation components and mitochondrial features, with adjusted *R* ^2^ values of 0.64 for Component 2 (*p*_FDR_ = 0.0005) and 0.47 for Component 4 (*p*_FDR_ = 0.01). Dominance analysis indicated that the spatial distributions of six mitochondrial features contributed equally to Component 2, while cytochrome c oxidase (CIV), total respiratory chain (TRC), and mitochondrial respiratory chain (MRC) were the primary contributors to Component 4.

## 4 Discussion

This study presents a novel framework integrating normative modeling and optimal nonnegative matrix factorization (opNMF) to characterize the heterogeneity of morphometric deviation patterns in self‐limited epilepsy with centrotemporal spikes (SeLECTS). Using opNMF, we decomposed morphometric deviations into eight distinct components with corresponding subject‐specific loadings. At the individual level, we related subject‐specific loadings to demographic and cognitive measures to identify morphometry‐phenotypic relationships. Furthermore, by integrating meta‐analytic functional decoding, neurotransmitter receptor distributions, and mitochondrial characteristics, we demonstrated that these components correspond to large‐scale cortical functions and molecular profiles.

The eight opNMF‐derived components identified in this study were highly localized and interpretable, reflecting distinct neuroanatomical patterns. Consistent with previous findings [33, 34], SeLECTS children exhibited thinner cortical thicknesses within the superior and inferior temporal gyrus and occipital regions, mainly in Components 1, 5, and 7. However, another study [35] showed thicker right superior frontal and superior temporal cortices and the larger amygdala volume in SeLECTS children, mainly in Components 4 and 5. Other studies [7] revealed thicker cortical thickness in the bilateral inferior frontal gyri and supramarginal gyrus, which spatial pattern was highly overlapped with Component 7. The brain regions related to changes in cortical thickness are concentrated in Components 1, 5, and 7, which are also the epileptogenic areas of the central temporal lobe. Component 2 exhibited pronounced values in subcortical structures, particularly the thalamus, while other components highlighted specific regions: Component 3 (caudate nucleus), Component 5 (amygdala), and Components 1, 6, and 8 (hippocampus). Studies have found that the volume of the caudate nucleus decreases [34], while others have found that the bilateral putamen and amygdala have increased [34, 35, 36, 37]. It is worth noting that the left and right hippocampus were decomposed into different components, which may be related to the asymmetry of the hippocampus [38, 39]. These findings indicate that morphometric deviation patterns in SeLECTS are expressed across multiple components, reflecting diverse neuroanatomical contributions rather than a single dominant pattern.

The bPLS analysis identified a significant latent variable (LV1) linking subject‐specific loadings to phenotypic profiles in SeLECTS. Consistent with our results of eight components (Figure 2A) and previous studies focusing on cortical thickness and subcortical volume [33, 34, 40], the components that significantly contributed to LV1 included 1, 3, 5, and 7. The spatial patterns of these components highly overlapped with the regions reported in the study where the two morphometric representations changed. The LV1 indicated associations between age, medication duration, disease duration, and full‐scale control score. A longitudinal study demonstrated that children with SeLECTS exhibited thinner cortex at baseline in frontal, temporal, and occipital regions (regions in Component 1 and 7) compared to controls. Over a 2‐year period, reduced cortical thinning was observed in the left rostral middle frontal gyrus, occipital gyrus, right superior frontal gyrus (regions in Component 1 and 7) in SeLECTS patients [34], suggesting that baseline anatomical characteristics may influence subsequent brain development. Notably, including age, the duration of medication and disease were all highly contributing characteristics to LV1, consistent with findings that the rate of cortical thickness changes with age were greater in SeLECTS than in controls [7]. Additionally, the contribution of scores of neuropsychological assessments (IQ scores and full‐scale comprehension, full‐scale control, and full‐scale attention) was all moderate. Considering the contribution of 10 subdimensions in the Figure S4, cautious and consistency dominated the full‐scale control, while focus dominated the full‐scale attention. It is crucial to distinguish between the two distinct aspects of validity affected by the missing data in our bPLS analysis. We acknowledge that the pattern of missingness, primarily driven by varying levels of participant compliance and recall accuracy, may limit the generalizability of our findings. Specifically, our results are most directly applicable to children with SeLECTS from families with high motivation and ability to complete extensive assessments. Caution should be exercised when extrapolating these findings to the broader population, particularly to those from families with lower compliance or poorer recall, who are under‐represented in our final analytical sample. However, we believe that the internal validity of the brain‐behavioral relationship identified in our cohort remains strong. Because there were no significant differences in measured clinical characteristics between the included subjects and those excluded due to data deficiency.

The meta‐analytic functional decoding of components that define the identified morphometry‐phenotypic LV (i.e., significant contributed component 3, 5 and 7 in LV1) match the cognitive processes that are believed to be disrupted during brain development, including emotion, cognitive control, working memory, and language, etc. [41, 42]. When exploring the associations between components and molecular profiles, the results of dominance analysis indicated that components (i.e., components 2, 3, and 5) strongly correlated with the distribution of neurotransmitters mainly involved functions such as emotion, decision‐making, and language. Previous studies have mainly shown through genetic variation analysis that the dysregulation of glutamate signaling in patients leads to the disruption of excitation‐inhibition balance, affecting these functions [43, 44]. More importantly, components 2 and 4 that contributed most to the distribution of mitochondrial features, and these components mainly involved in functions related to emotion and language. Mitochondrial dysfunction and seizures have been found to be closely linked not only in genetic causes of epilepsy, but are also becoming increasingly recognized in acquired epilepsy [45]. The interaction between epilepsy and mitochondrial dysfunction depletes the energy storage of neurons, leading to extensive brain damage.

While the SeLECTS clinical cohort exhibits a different age distribution from the devCCNP normative cohort (Table 1), our modeling approach inherently addresses this concern. The multivariate fractional polynomial regression (MFPR) algorithm generates individualized, age‐specific predictions for each participant, ensuring that calculated *z*‐scores represent deviations from demographically adjusted expectations rather than cohort‐level averages. The robustness of this approach is empirically supported by the CentileBrain framework, which demonstrated stable model accuracy across distinct age bins. This methodological strength transforms the intercohort age difference into a validation of model generalizability. The significant neuroanatomical deviations identified in the SeLECTS cohort thus likely reflect true clinical characteristics rather than artifacts of population age structure, enhancing the validity of our findings.

## 5 Limitations

This study has several limitations that warrant consideration. First, the sample size of children with SeLECTS was relatively small, which may limit the stability and generalizability of the normative models. A larger sample could enhance the robustness of the findings. Second, site‐related effects remain a challenge when applying trained models to data from new sites. Although we assessed the generalizability of the model by examining morphometric deviations in healthy controls from the SeLECTS dataset, individual deviations exceeded the 5% threshold in a subset of brain regions, such as those surrounding the central sulcus. Third, there was a difference in age distribution between the SeLECTS and devCCNP queues. The standardized modeling method reduces the influence of age differences in methodology. The MFPR algorithm generates personalized, age‐specific predictions to ensure that the z‐score reflects the deviation of age‐adjusted expectations rather than differences at the group level. This method has been empirically verified by the CentileBrain framework, which demonstrates the consistent accuracy of the model across different age ranges. Forth, the choice of brain atlas influenced the results, as the number and delineation of brain regions vary across atlases. Parcellations based on cytoarchitecture or functional connectivity may differ in sensitivity compared to conventional cortical thickness modeling, potentially affecting the observed morphometric patterns. Fifth, while we included age and disease duration as covariates in our bPLS analysis to account for some heterogeneity, a cross‐sectional design is inherently limited in its ability to trace the trajectory of neuroanatomical deviations. It cannot directly observe whether the identified structural bias is a transient state marker that fades with clinical remission, a persistent effect of the disease, or even a predictor of long‐term cognitive outcomes. A longitudinal design is undoubtedly the gold standard for elucidating the clinical significance of these findings within the natural course of SeLECTS.

## 6 Conclusion

This work combined normative modeling and nonnegative matrix factorization to investigate subject‐level variation that links brain and behaviors across brain morphometric measures. Our study identified eight deviation patterns in SeLECTS with increased and decreased cortical thicknesses or subcortical volumes, indicating that individual heterogeneity is primarily reflected in the different compositions of these components. These patterns were associated with distinct neurotransmitter systems and mitochondrial features. Further, this work could be applied in future studies of brain development and in the context of neurological disorders to parse heterogeneity.

## Author Contributions

Conceptualization, supervision, project administration and funding acquisition, Z.Z. Formal analysis and methodology, J.X. Resources and software: W.L. Visualization, C.P. Validation, Y.Z. and J.W. Writing – original draft, S.Y. Writing – Review and Editing, S.Y.

## Funding

This work was supported by National Natural Science Foundation of China, 82302293 and 82371951. Sichuan Natural Science Foundation, 2024NSFSC1782. National Science and Technology Innovation 2030‐Neuroscience and Brainlike research, 2022ZD0211800. Xuzhou Medical University Affiliated Hospital Development Fund Project, XYKF202101.

## Ethics Statement

The study has been approved by the medical ethics committee of Jinling Hospital, School of Medicine, Nanjing University, China (approval number: 2018NZKY‐020‐02). Written informed consent was obtained from all participants in accordance with the Declaration of Helsinki.

## Conflicts of Interest

The authors declare no conflicts of interest.

## Supporting information


**Data S1:** cns70794‐sup‐0001‐supinfo.docx.

## Data Availability

The datasets used and/or analyzed during the current study are available from the corresponding author on reasonable request.
