# Supplementary material for: Linking Cortical Morphometry in Self‐Limited Epilepsy With Centrotemporal Spikes to Cognition, Function, and Molecular Architecture
Source: CNS Neurosci Ther. 2026 Feb 23;32(2):e70794. doi: 10.1002/cns.70794 (PMC12928023; doi:10.1002/cns.70794)
Supplement: Supplementary file 1 — Data S1: cns70794‐sup‐0001‐supinfo.docx. [file CNS-32-e70794-s001.docx]

Supplementary Information for:

**Linking cortical morphometry in** **Self-limited epilepsy with Centrotemporal Spikes to cognition, function, and molecular architecture**

**Siqi Yang^1^, Jie Xia^2^, Wei Liao^3^, Yimin Zhou^1^, Chengzong Peng****^1^, Juan Wang^1^, Zhiqiang Zhang^4^**^🖂^

^1^ School of Cybersecurity (Xin Gu Industrial College), Chengdu University of Information Technology, Chengdu, 610225, P.R. China.

^2^ School of Acupuncture and Tuina, Chengdu University of Traditional Chinese Medicine, Chengdu 611137, P.R. China.

^3^ The Clinical Hospital of Chengdu Brain Science Institute, School of Life Science and Technology, University of Electronic Science and Technology of China, Chengdu, 610054, P.R. China.

^4^ Lab. of Neuroimaging, Dept. of Radiology, Jinling Hospital, Nanjing University School of Medicine, Nanjing, 210002, P.R. China.

^🖂^ Corresponding authors:

Zhiqiang Zhang (zhangzq2001@126.com)

Department of Medical Imaging, Nanjing Jinling Hospital, 305#, Eastern Zhongshan Rd.,

Nanjing 210002, PR China. Fax: +86-25-84804659. Tel: +86-25-84804659.

CONTENTS

[Supplemental Methods 3](#_Toc215681734)

[Supplemental Tables 5](#_Toc215681735)

[Supplemental Figures 9](#_Toc215681736)

**Supplemental Methods**

**The potential effect of site**

**Normative modeling was performed using multi-center data, whereas the SELECTS cohort was acquired from a single site. To evaluate whether the deviations from the normative model observed in SELECTS individuals reflected site-related differences, we calculated deviation values (i.e., z-scores) in healthy control data from the SeLECTS dataset. We first computed global average morphometric deviations (z-scores) across the whole brain, and examined region-specific morphological deviations, as shown in *Fig.S1*. As shown in Fig.S1-A, the mean z-scores for cortical regions exhibiting below or above the normative range in cortical thickness were -0.41/0.42, while the corresponding values for subcortical volume were -0.38/0.44. *Fig.S1-B* and *Fig.S1-C* illustrate the distribution of z-scores for specific subcortical structures and cortical regions, respectively, within the normal control group. Z-scores below –1.96 or above 1.96 were considered to represent infra-normative or supra-normative deviations, respectively. *Fig.S2* have shown that more than 95% of individual deviations across most brain regions fell within the normative range, indicating that the normative model trained in this study exhibits satisfactory generalizability when applied to data from new sites.**

**The scanner and parameters for the SeLECTS cohort are largely consistent with those of the CKG sample, as both utilized 3.0-T Siemens Trio MRI scanner. The PEK sample employed a different scanner model, 3.0-T GE Discovery MR750 scanner. To mitigate the confounding effects of multi-site data acquisition and differing scanning parameters, we implemented a proactive harmonization step using the ComBat-GAM. This data-driven method was applied to the neuroimaging data from the multi-site normative cohort (devCCNP) prior to normative model training. ComBat-GAM effectively removes site-specific biases by estimating and adjusting for additive and multiplicative scanner effects within the data. Subsequently, the harmonization parameters derived from the normative cohort training data were applied to the single site SeLECTS cohort data. This process projects the clinical cohort data into the same harmonized feature space as the normative sample, ensuring that subsequent comparisons of individual deviations (z-scores) are not systematically biased by differences in acquisition protocols between the cohorts.**

**Supplemental Tables**

**Table S1.** Neuropsychological assessments.

| assessments | scores  (mean ± std) |
| --- | --- |
|  |  |
| IQ score | 105.8 ± 21.1 |
| full-scale comprehension | 86.9 ± 17.2 |
| full-scale control | 75.2 ± 23.6 |
| full-scale attention | 78.2 ± 24.4 |
| hyperactivity | 99.0 ± 12.2 |
| cautious | 71.7 ± 36.3 |
| consistency  perseverance | 79.8 ± 27.3 |
|  | 95.5 ± 22.7 |
| alertness | 91.7 ± 122.1 |
| focus | 73.9 ± 33.8 |
| speed | 105.8 ± 118.2 |
| balance | 136.2 ± 27.5 |
| agility | 103.9 ± 118.5 |
| sustainability | 113.2 ± 78.3 |
| sensorimotor | 102.0 ± 31.7 |

**Table S2.** Latent variables identified by bPLS (n=38).

| Latent Variable (LV) | singular value | variance explained | *p*-value |
| --- | --- | --- | --- |
| LV 1 | 1.01 | 30% | 0.01* |
| LV 2 | 0.82 | 25% | 0.21 |
| LV 3 | 0.49 | 15% | 0.49 |
| LV 4 | 0.37 | 11% | 0.52 |
| LV 5 | 0.32 | 10% | 0.92 |
| LV 6 | 0.18 | 5% | 0.99 |
| LV 7  LV 8 | 0.10 | 3% | 0.99 |
|  | 0.04 | 1% | 0.99 |

* represented significant *p*-value.

**Table S3.** Latent variables from the brain-cognitive bPLS analysis.

| Latent Variable (LV) | singular value | variance explained | *p*-value |
| --- | --- | --- | --- |
| LV 1 | 0.76 | 25% | 0.26 |
| LV 2 | 0.64 | 22% | 0.02* |
| LV 3 | 0.49 | 16% | 0.52 |
| LV 4 | 0.36 | 12% | 0.68 |
| LV 5 | 0.27 | 9% | 0.88 |
| LV 6 | 0.20 | 7% | 0.99 |
| LV 7  LV 8 | 0.15 | 5% | 0.99 |
|  | 0.10 | 3% | 0.99 |

* represeted significant *p*-value.

**Table S4.** Latent variables from the brain-clinical bPLS analysis.

| Latent Variable (LV) | singular value | variance explained | *p*-value |
| --- | --- | --- | --- |
| LV 1 | 0.52 | 33% | 0.02* |
| LV 2 | 0.38 | 24% | 0.16 |
| LV 3 | 0.34 | 22% | 0.17 |
| LV 4 | 0.16 | 10% | 0.97 |
| LV 5  LV 6 | 0.12 | 8% | 0.98 |
|  | 0.05 | 3% | 0.99 |

* represeted significant *p*-value.

**Supplemental Figures**

**Fig.S1**

**Fig.S1** Global averaged z-scores and region-specific z-scores of the trained normative model applied on the healthy control group of the SeLECTS dataset. To verify the robustness of the model's performance, we calculated the z-scores of the trained normative model on the healthy control group of the SeLECTS dataset. (A) Prediction error was evaluated by calculating the mean absolute deviation of each healthy subject across the 68 cortical regions /14 subcortical structures. Blue represents below the normative range, and yellow represents above the normative range. The distribution of z-scores for (B) each subcortical structure and (C) each cortical region in the healthy control group. The dotted vertical lines represent the cutoffs for z-scores < −1.96 and z-scores >1.96 values. Brain morphometric measures with values below the 5^th^ percentile or above the 95^th^ percentile of the normative range were respectively considered infra-normal and supra-normal. Lightblue represents left hemisphere, and orange represents right hemisphere.

**Fig.S2**

**Fig.S2.** Percentage of participants with infra- and supra-normal deviations. **(A)** Patterns of infra-normal supra-normal deviations in subcortical volume and **(B)** cortical thickness in healthy controls of SeLECTS dataset.

**Fig.S3**

**^^**

**Fig.S3** Stability coefficient (blue curve) and gradient of the reconstruction error (red curve) for solutions of k=2 to k=20. The yellow box indicates that k=8 selected for further analysis.

**Fig.S4**

**^^**

**Fig.S4** **Latent morphometry-cognitive relationships identified by bPLS. (A)** Scree plot of the singular value (blue dot) and covariance explained (red asterisk) by all latent variables (LVs) in the bPLS analysis, with LV2 significant at *p* = 0.02. **(B)** Bar chart illustrating the loadings of neuropsychological test scores contributing to LV2. Bar height represents the loading magnitude, with error bars indicating the 95% confidence interval derived from bootstrap resampling. Blue bars denote statistically significant features. **(C)** Bar plot of bootstrap ratios (BSRs) for each component.

**Fig.S5**

**^^**

**Fig.S5** **Latent morphometry-clinical relationships identified by bPLS. (A)** Scree plot of the singular value (blue dot) and covariance explained (red asterisk) by all latent variables (LVs) in the bPLS analysis, with LV1 significant at *p* = 0.02. **(B)** Bar chart illustrating the loadings of clinical variables contributing to LV1. Bar height represents the loading magnitude, with error bars indicating the 95% confidence interval derived from bootstrap resampling. Blue bars denote statistically significant features. **(C)** Bar plot of bootstrap ratios (BSRs) for each component.

**Fig.S6**

**^^**

**Fig.S6** Correlations between the predicted deviation components and the empirical deviation components in the training set **(A)** and test set (**B**). All multilinear models between deviation components and receptor/transporter density distributions were cross-validated using distance-dependent methods. This method selects 25% of the regions closest to the source region as the training set, and the remaining 75% of the regions as the test set. Each brain region is used as the source region to repeat this process (100 iterations). The circles in the violin diagram represent the median, and the lines span the non-contour minimum and maximum values of the distribution.

**Fig.S7**

**Fig.S7** Correlations between the predicted deviation components and the empirical deviation components in the training set (A) and test set (B). All multilinear models between deviation components and mitochondrial features distributions were cross-validated using distance-dependent methods.
